# Multi-Tissue Transcriptome Profiling of North American Derived Atlantic Salmon

**DOI:** 10.3389/fgene.2018.00369

**Published:** 2018-09-13

**Authors:** Amin R. Mohamed, Harry King, Bradley Evans, Antonio Reverter, James W. Kijas

**Affiliations:** ^1^Commonwealth Scientific and Industrial Research Organisation Agriculture and Food, Queensland Bioscience Precinct, St Lucia, QLD, Australia; ^2^Zoology Department, Faculty of Science, Benha University, Benha, Egypt; ^3^Commonwealth Scientific and Industrial Research Organisation Agriculture, Hobart, TAS, Australia; ^4^Tassal Ltd., Hobart, TAS, Australia

**Keywords:** Atlantic salmon, RNA-Seq, transcriptome, gene expression, transcription factors, next-gen sequencing

## Abstract

The availability of a reference genome assembly for Atlantic salmon, *Salmo salar*, SNP genotyping platforms and low cost sequencing are enhancing the understanding of both life history and production-related traits in this important commercial species. We collected and analyzed transcriptomes from selected tissues of Atlantic salmon to inform future functional and comparative genomics studies. Messenger RNA (mRNA) was isolated from pituitary gland, brain, ovary, and liver before Illumina sequencing produced a total of 640 million 150-bp paired-end reads. Following read mapping, feature counting, and normalization, cluster analysis identified genes highly expressed in a tissue-specific manner. We identified a cluster of 508 tissue specific genes for pituitary gland, 3395 for brain, 2939 for ovary, and 539 for liver. Functional profiling identified gene clusters describing the unique functions of each tissue. Moreover, highly-expressed transcription factors (TFs) present in each tissue-specific gene cluster were identified. TFs belonging to *homeobox* and *bhlh* families were identified for pituitary gland, *pou* and *zf-c2h2* families for brain, *arid*, and *zf-c2h2* for ovary and *rxr*-like family for liver. The data and analysis presented are relevant to the emerging Functional Annotation of All Salmonid Genomes (FAASG) initiative that is seeking to develop a detailed understanding of both salmonid evolution and the genomic elements that drive gene expression and regulation.

## Introduction

The Atlantic salmon (*Salmo salar*), a member of the family Salmonidae, is endemic to the northern Atlantic Ocean and cultivated worldwide including in Tasmania, Australia. The Australian salmon industry is founded on introduced North American wild stock originating from the River Philip in Nova Scotia (Jungalwalla, 1991). To enhance productivity using genetics, an applied breeding program has been in operation since 2004, and the Tasmanian salmon industry is now the highest valued commercial fishery-related industry in the state (Australian Bureau of Agricultural Resource Economics and Sciences, 2014). A number of production issues remain which are potentially tractable using genomic approaches, including unwanted early maturation, disease susceptibility, product quality traits, and imperfect DNA based diagnostics for sex determination.

The ability to elucidate the basis of complex salmonid traits has been dramatically improved by the availability of reference genomes for both rainbow trout (*Oncorhynchus mykiss*) (Berthelot et al., 2014) and Atlantic salmon (Lien et al., 2016). To date, their major impact has been through enabling the development and application of high density SNP genotyping platforms and GWAS. The availability of annotated protein coding gene models supports the interpretation of GWAS to identify putatively causal variants contributing to trait variation (Ayllon et al., 2015; Barson et al., 2015). However, these reference genomes currently lack annotation of the sequences that regulate gene expression, including proximal, and distal promotors and enhancer elements. Functional annotation requires significant time and cost, and the animal science community has self-assembled into consortia to tackle the task for livestock genomes (FAANG consortium, Andersson et al., 2015) and salmonids inside the “Functional Annotation of All Salmonid Genomes” (FAASG, Macqueen et al., 2017). The experimental component involves collection of multiple data types to support functional annotation, including histone modification marks, DNA methylation status and gene expression. RNA-Seq has revolutionized transcriptomic research, previously based on testing expression of candidate genes using RT-qPCR or even thousands of genes using cDNA microarrays. RNA-Seq provides an efficient and unbiased tool for large scale gene expression analyses in non-model organisms (for example Mohamed et al., 2016, 2018) as well as in salmonids (Salem et al., 2015; Kim et al., 2016; Robinson et al., 2017). Additional background information about available genomic tools for salmonid research, in the broader context of aquaculture genomics, is captured in a recent review (Abdelrahman et al., 2017).

The objective of this study was to begin contributions to FAASG by transcriptomic profiling selected tissues. We prioritized the brain-pituitary-gonad (BPG) axis, which plays a critical role in the development and regulation of the reproductive and immune systems in vertebrates including teleosts (Weltzien et al., 2004; Cowan et al., 2017). Fluctuations in this axis regulate hormone production, which exert local and systemic effects on the animal relating to sexual development, maturation, and other traits. The liver is an essential metabolic organ as it regulates most chemical levels in the blood, detoxifies various metabolites, synthesizes proteins, and produces energy. Hence, analyzing the complement of expressed genes within and across these four tissues will provide a basis for understanding key production and life history traits.

## Materials and methods

### Ethics statement

The animals used are from the SALTAS selective breeding program which has been described previously (Dominik et al., 2010; Eisbrenner et al., 2014; Kijas et al., 2017). The four animals used were sexually immature 3 year old female fish from the 2014 year class. The fish were reared and euthanized in compliance with the *CSIRO Animal Ethics Committee*, under approval number 2017–02.

### Tissue collection and total RNA isolation

Samples from mid-brain, pituitary gland, ovary and liver were collected and stored in RNA-later at −80°C until RNA isolation. Total RNA was isolated from each tissue using RNeasy Kit (QIAGEN), eluted in 40 μL RNase-Free Water and stored at −80°C. RNA quantity were assessed using a Nanodrop™ ND-1000 spectrophotometer (Thermo Scientific), and integrity checked by electrophoretic profiling with Agilent Bioanalyzer 2100 (Agilent, CA).

### High-throughput mRNA sequencing (illumina RNA-Seq)

Messenger RNA (mRNA) was isolated from 500 ng total RNA and a total of 16 RNA-Seq libraries (4 tissues × 4 biological replicates per tissue) were prepared using the Illumina TruSeq stranded library Preparation Kit. Libraries were sequenced on an Illumina NovaSeq 6,000 sequencing platform producing 640.3 million individual 150-bp paired-end reads.

### Read mapping, differential gene expression, and clustering analyses

Illumina reads were checked for quality using FastQC software. High quality reads (Q>30) were mapped to Atlantic salmon genome ICSASG_v2 (Lien et al., 2016) using TopHat2 version 2.1.1 (Kim et al., 2009) with default parameters. Alignment BAM files were sorted by read name and converted into SAM files using SAMtools version 1.4 (Li et al., 2009). The Python package HTSeq version 0.7.2 (Anders et al., 2015) was applied to count unique reads mapped to exons, which is suitable for RNA-Seq analysis. Raw counts were analyzed using the edgeR package (Robinson et al., 2010) in the R statistical computing environment to infer differential gene expression among tissues. To identify differential expression between samples and tissues, we applied both fold-change and false discovery rate (FDR) thresholds. Specifically, we set FDR to ≤ 0.001 and log_2_ (fold change) ≥2 to declare significance in line with previous recommendations (Haas et al., 2013). It is important to note a log_2_ (fold change) >2 equates with a minimum 4-fold change. To compare multiple expression profiles, we used the value of log_2_ (FPKM+1) centered by subtracting the mean across profiles (Haas et al., 2013), i.e. the mean-centered log_2_ (FPKM+1) value in which the FPKM+1 circumvents the non-definable instances of log_2_(0) if FPKM = 0. Tissue-specific gene clusters were identified as those exhibiting high expression in one tissue versus all others.

### Gene ontology (GO) enrichment analysis of the tissue-specific gene clusters

To infer function for identified tissue-specific clusters, GO enrichment analyses were performed using the Database For Annotation, Visualization And Integrated Discovery (DAVID) (Huang et al., 2009). The ENTREZ_GENE_ID of the gene clusters was used as identifier and *Salmo salar* was selected as the background dataset for the enrichment analysis. DAVID uses Fisher's exact test to ascertain statistically significant enrichment of pathways amongst differentially expressed genes (DEGs) relative to the background transcriptome. For the purpose of the enrichment analysis, GO categories with a Benjamini-corrected *P* ≤ 0.05 were considered significant (Huang et al., 2009). The RNA-Seq reads used in this study have been submitted to the NCBI Gene Expression Omnibus (GEO) database under Accession no. GSE114247.

### Identification of highly expressed transcription factors (TFs)

In order to determine transcription factors (TFs) in the tissue-specific gene clusters in Salmon, BLASTX analysis were performed (*E*-value < 10^−3^) against the Cod annotated transcription factors database downloaded from the AnimalTFDB database (http://bioinfo.life.hust.edu.cn/AnimalTFDB/#!/tf_summary?species=Gadus_morhua) for *Gadus morhua*. The retrieved transcription factors were ranked based on their expression in each of the tissue-specific clusters.

## Results

### Expression within and between four selected tissues

Transcriptomic data was collected from 16 RNA-Seq libraries constructed in a 4 (tissues) × 4 (replicates) experimental design. Sequencing yielded a total of 640 M raw reads, which after mapping to the Atlantic salmon reference assembly ICSASG_v2 yielded an average of 28 M reads per library (Table S1). To begin assessment between libraries, multidimensional scaling (based on the top 500 genes that best differentiate the samples) was performed (Figure S1). This confirmed biological replicates within tissue clustered together, and libraries from different tissues took non-overlapping positions. We performed hierarchical clustering using Spearman correlations of pairwise normalized expression for all 16 samples to explore the relative amount of variation between replicates within a tissue compared to that between the four tissues. This revealed that brain displayed the highest variation between the four biological replicates (animals) while the lowest was observed for ovary tissue. Further, clustering grouped the brain and pituitary as the two tissues with the most similar patterns of gene expression (Figure S2). Together these assessments indicated gene expression variability was much lower between replicates of the same tissue as compared with variation between tissues, as expected for a high quality dataset.

### Gene clusters with tissue specific expression

**Figure 1:**
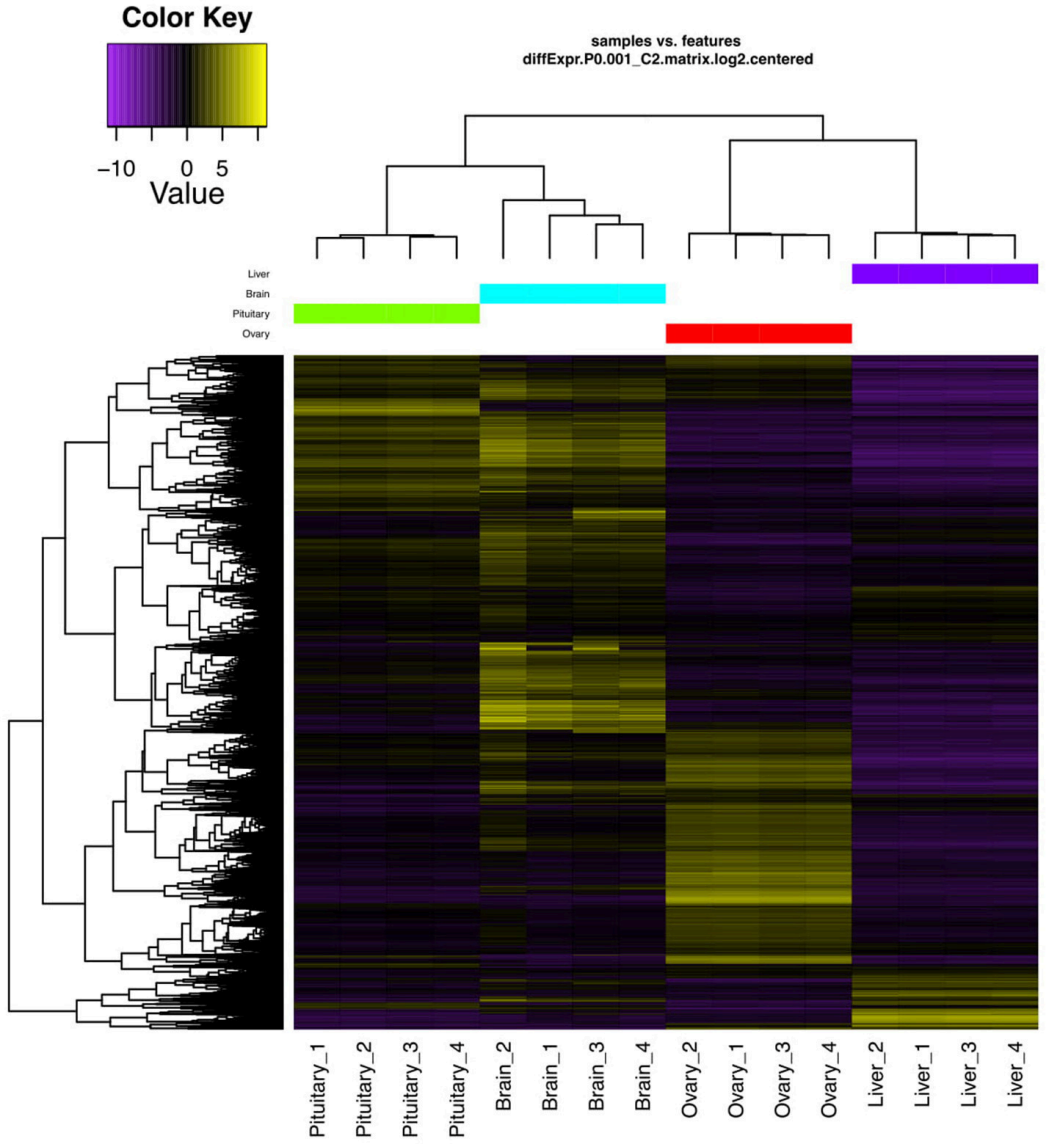
DEGs hierarchical clustering. Heat map showing genes (rows) with differential expression (fold ≥ 4, FDR ≤ 0.001) among the four replicates of the pituitary gland, brain, ovary, and liver samples. Expression values are log_2_-transformed and median-centered by gene.

**Figure 2:**
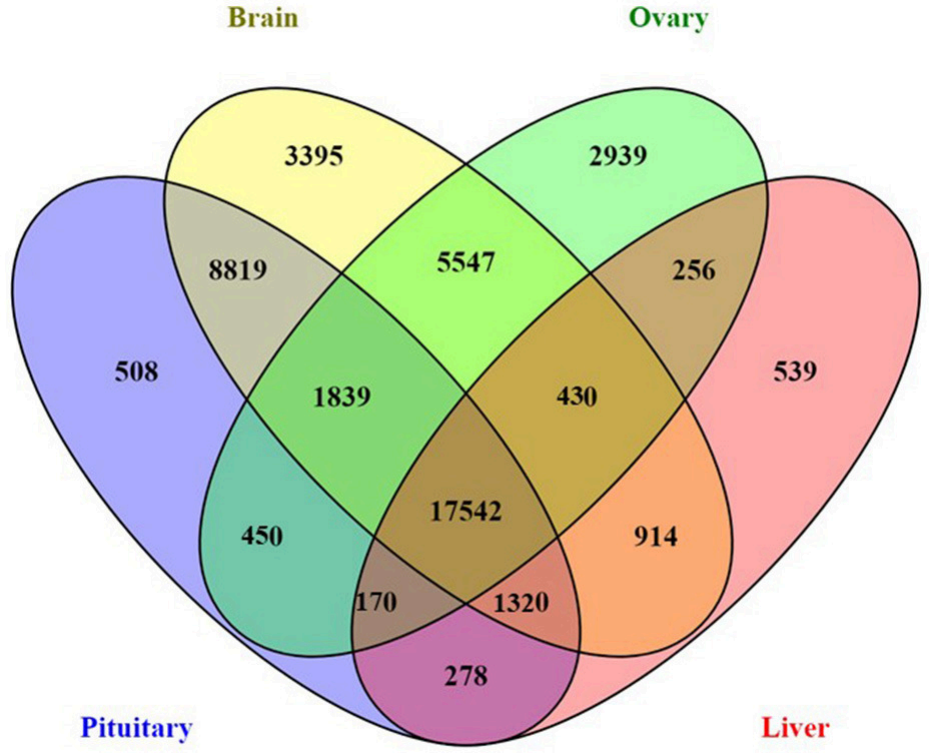
Four-way Venn diagram showing the number of shared and unique expressed genes across four salmon tissues (pituitary gland, brain, ovary, and liver).

Genes exhibiting significant differential expression (DEG, ≥4-fold change with false discovery rate FDR ≤ 0.001) were identified and used to search for gene clusters exhibiting tissue specific expression. Hierarchical clustering using only significant DEGs revealed distinctive expression profiles for each tissue (Figure 1). Brain appeared to have the highest occurrence of tissue specific expression (3395 genes) followed by ovary (2939), liver (539), and the pituitary gland (508) (Figure 2). Apart from 17542 genes that were shared among the four tissues, the highest number of shared genes were 8819 between pituitary gland and brain followed by 5547 between brain and ovary, and interestingly 1839 among pituitary gland, brain and ovary (Figure 2). This can be expected given the important role of the brain-pituitary-gonad (BPG) axis in regulating sexual development in vertebrates including fish.

**Figure 3:**
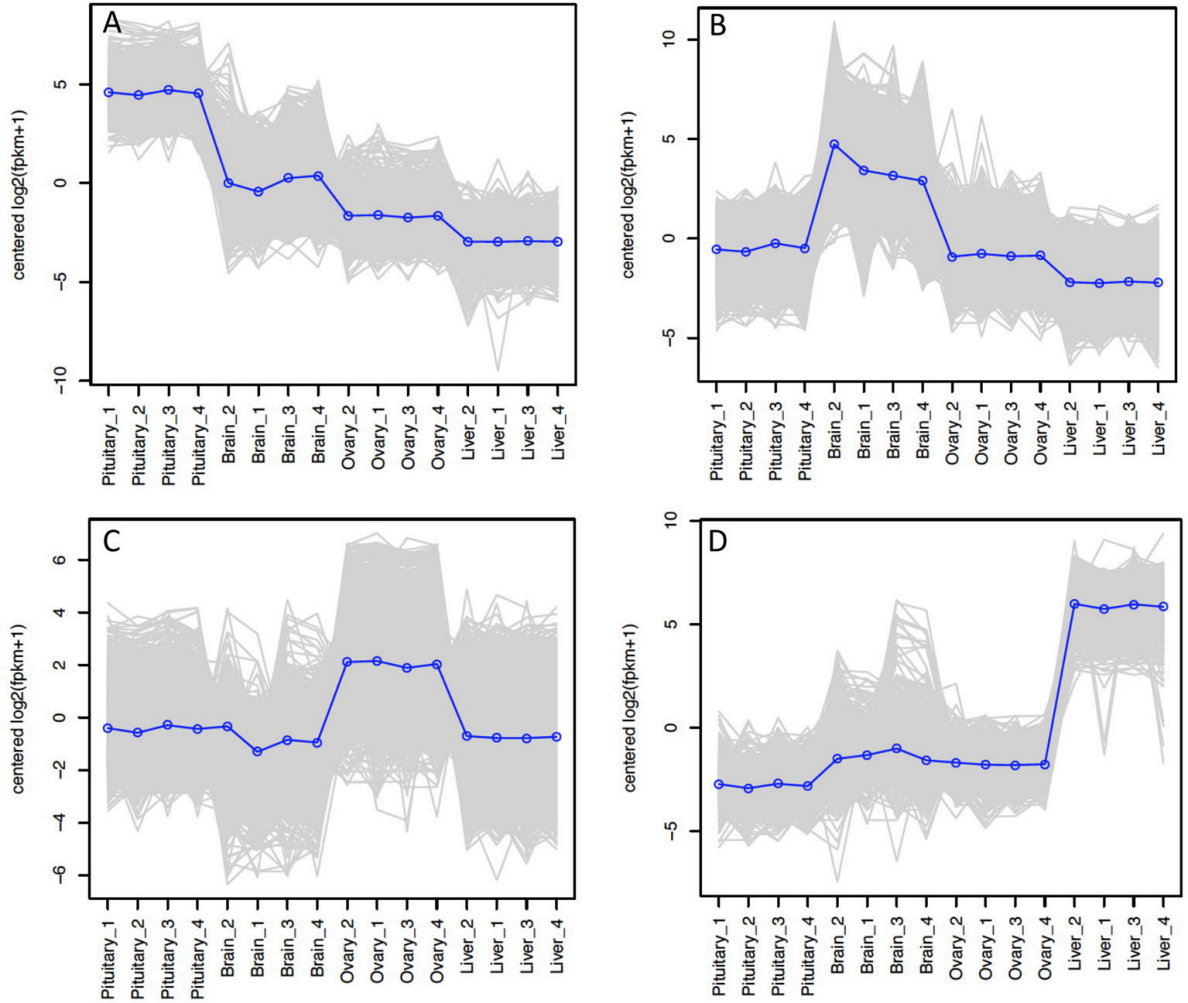
Tissue-specific clusters in Atlantic Salmon from Tasmania. Cluster of 508 genes was identified for pituitary gland **(A)**, 3395 for brain **(B)**, 2939 for ovary **(C)**, and 539 for liver **(D)**. The y-axis in each graph represents the mean-centered log2 (FPKM+1) value. Expression of single genes is plotted in gray, while the mean expression of the genes in each cluster is plotted in blue.

**Figure 4:**
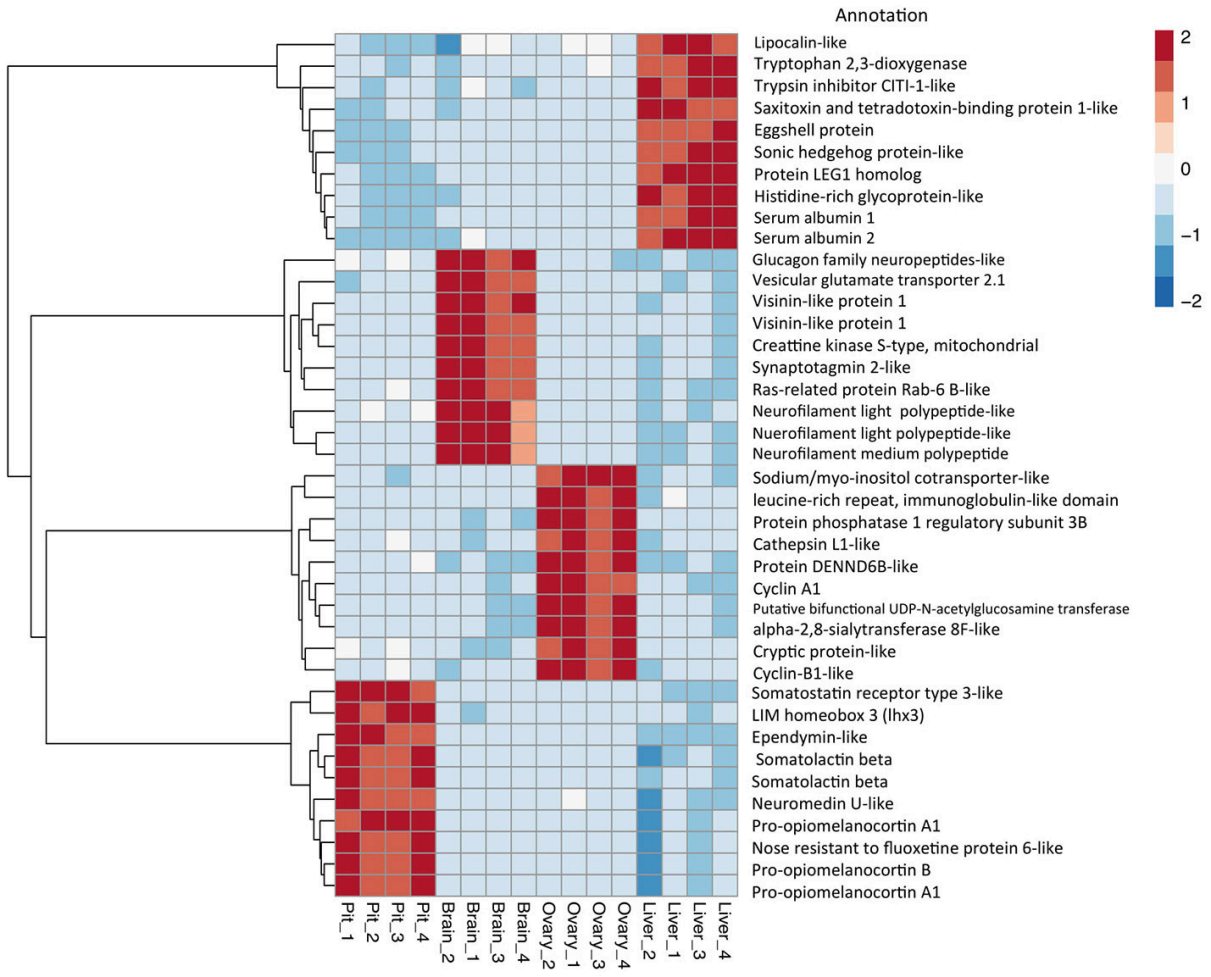
Top 10 differential expressed gene expression (DEGs). Heat map and dendrogram of the 10 most highly expressed genes in each tissue-specific cluster. The red-blue spectrum represents the scaled expression values.

The relative expression of the identified tissue specific clusters is compared across tissues in Figure 3. We sought to explore if tissue specific gene clusters were enriched for gene ontologies (GO) relating to molecular function (GO-MF), cellular component (GO-CC), or biological process (GO-BP). Pituitary gland-specific genes contained significant enrichment for all three gene ontology classes (GO-BP, GO-CC GO-MF terms at corrected *P* ≤ 0.05, Table 1). Despite being comprised of only three genes (*growth hormone pre-peptide, prolactin, somatotropin-2*), the most highly over-represented term (149 fold) was *regulation of immunoglobulin secretion* that includes genes coding for growth hormones, prolactin, and somatotropin. The GO-CC *extracellular region* was identified via pituitary gland hormones such as *somatolactins, thyroid stimulating hormone, pro-opiomelanocortin*, and *gonadotropin* which is consistent with the expected gene expression profile of the pituitary (Table 1, Table S3). The list of highly expressed genes in the pituitary gland included genes with function related to hormone activity such as *somatolactin beta, pro-opiomelanocortin A1* and *B* (Figure 4; Table S2). The list of highly expressed transcription factors (TFs) identified in the pituitary gland (Table 2) included *lhx3, six1b*, other homeodomain proteins, *ascl1a, bhlha15, zf-c2h2*, and *zbtb*. Brain-specific genes were enriched for the GO-CC *extracellular region* term due to expression of neuropeptides including *proenkephalin, myostatins, cholecystokinins*, and *thyrotropin-releasing hormone* (Table 1, Table S4). Further, GO-MF term *calcium ion binding* was enriched based on expressed neuronal calcium-sensor/binding proteins such as *visinin-like proteins, calretinin-like proteins* and *calmodulins* (Table 1; Table S4). The list of highly expressed genes in the brain (Figure 4; Table S2) included *Vesicular glutamate transporter 2.1* (gene ID = gene48716:106587477) and several genes coding for neuropeptides such as *Glucagon family neuropeptides-like* (gene ID = gene39762:106578701). The list of highly expressed TFs identified in the brain included *zic2a, zic5, pou3f2a, pou3f3b, esrrb* (Table 2).

**Table 1:** Gene Ontology (GO) categories enriched (corrected *P* ≤ 0.05) among tissue-specific gene clusters.

**Tissue**	**Category**	**GO term ID**	**GO term description**	**Gene count**	**Fold enrichment**
Brain	GO_CC	GO:0005576	Extracellular region	21	3.56
	GO_MF	GO:0005509	Calcium ion binding	17	2.86
Pituitary	GO_BP	GO:0051023	Regulation of immunoglobulin secretion	3	149.66
		GO:0050766	Positive regulation of phagocytosis	3	112.25
		GO:0032930	Positive regulation of superoxide anion generation	3	74.83
	GO_CC	GO:0005576	Extracellular region	11	7.18
	GO_MF	GO:0043565	Sequence-specific DNA binding	7	4.13
Ovary	GO_CC	GO:0005730	Nucleolus	9	5.39
		GO:0016021	Integral component of membrane	115	1.31
	GO_MF	GO:0016853	Isomerase activity	6	9.05
Liver	GO_BP	GO:0006869	Lipid transport	4	51.31
		GO:0042157	Lipoprotein metabolic process	4	44.9
	GO_CC	GO:0005576	Extracellular region	11	6.47
		GO:0005615	Extracellular space	6	7.03
	GO_MF	GO:0008289	Lipid binding	6	11.11

*Significant enrichment for biological process (GO_BP), cellular component (GO_CC) and molecular function (GO_MF) are indicated along with their term descriptors. The identity of the genes driving each enrichment are provided in the Supplementary material*.

**Table 2:** Transcription factors highly expressed (top 10 ranked) in each tissue-specific gene clusters along with their expression.

**Salmon gene ID**	**ENSB ID**	**Gene symbol**	**Family**	**Description**	**Pituitary**	**Brain**	**Ovary**	**Liver**
gene29567:106568496	ENSGMOG00000003038	*znf532*	*zf-c2h2*	Zinc Finger Protein 532	7.076638	−1.81893	−1.75861	−3.4991
gene16311:106609474	ENSGMOG00000002268	*ascl1a*	*bhlh*	Achaete-scute homolog 1	6.812102	−1.07506	−2.32828	−3.40876
gene15527:106608660	ENSGMOG00000007895		*homeobox*	Homeodomain protein	6.511932	−1.66275	−1.85147	−2.99771
gene24096:106563263	ENSGMOG00000015270	*lhx3*	*homeobox*	LIM homeobox 3	6.489042	−2.28645	−0.13382	−4.06877
gene38047:106576936	ENSGMOG00000000185		*homeobox*	Homeodomain protein	6.357463	−1.26608	−0.20003	−4.89135
gene37389:106576285	ENSGMOG00000002268	*ascl1a*	*bhlh*	Achaete-scute homolog 1	6.204624	−0.17073	−2.43396	−3.59994
gene51125:106589580	ENSGMOG00000003972	*bhlha15*	*bhlh*	Basic Helix-Loop-Helix Family Member A15	6.181782	−1.24124	−2.72763	−2.21291
gene1631:106610965	ENSGMOG00000006624		*homeobox*	Homeodomain protein	6.168265	0.592351	−2.62659	−4.13403
gene32739:106571735	ENSGMOG00000009091	*zbtb49*	*zbtb*	zinc finger and BTB domain containing 49	6.054175	0.013972	−2.49383	−3.57431
gene598:106598867	ENSGMOG00000010826	*six1b*	*homeobox*	Homeobox protein six1b	5.780814	0.274847	−2.0676	−3.98806
gene14956:106608144	ENSGMOG00000009083	*myt1a*	*zf–c2hc*	Myelin Transcription Factor 1	−1.56162	6.430779	−2.08082	−2.78834
gene48129:106586884	ENSGMOG00000018584	*olig2*	*bhlh*	Oligodendrocyte Transcription Factor 2	−1.55954	6.344058	−1.78626	−2.99826
gene14733:106607949	ENSGMOG00000007407	*pou3f2a*	*pou*	POU class 3 homeobox 2a	−1.51858	6.216699	−2.34906	−2.34906
gene42796:106581638	ENSGMOG00000019585	*zic2a*	*zf-c2h2*	zic family member 2a	−1.31137	6.161513	−1.80161	−3.04854
gene47289:106586041	ENSGMOG00000019582	*zic5*	*zf-c2h2*	Zic Family Member 5	−1.51915	6.075141	−1.75899	−2.79699
gene34430:106573296	ENSGMOG00000004267	*rfx4*	*rfx*	Regulatory Factor X4	−1.38598	6.045839	−0.24805	−4.4118
gene35702:106574614	ENSGMOG00000015654	*pou3f3b*	*pou*	POU class 3 homeobox 3b	−0.36971	6.030068	−2.43394	−3.22642
gene17894:106611051	ENSGMOG00000015180	*esrrb*	*esr-like*	Steroid hormone receptor ERR2	−1.56374	5.9837	−2.20998	−2.20998
gene4377:106584047	ENSGMOG00000005989	*pou3f2b*	*pou*	POU class 3 homeobox 2b	−1.46208	5.928734	−2.10832	−2.35832
gene11670:106605033	ENSGMOG00000005989	*pou3f2b*	*pou*	POU class 3 homeobox 2b	−2.10764	5.926683	−1.4614	−2.35764
gene50315:106588731	ENSGMOG00000013263		*thap*	THAP domain containing	−1.75755	−1.61131	5.62642	−2.25755
gene31170:106569971	ENSGMOG00000001476	*kdm5ba*	*arid*	Lysine (K)–specific demethylase 5Ba	−1.22916	−1.91391	5.570603	−2.42752
gene45968:106584708	ENSGMOG00000002555	*arid3b*	*arid*	AT-Rich Interaction Domain 3B	0.31235	−2.44804	5.494693	−3.359
gene22766:106561922	ENSGMOG00000019253	*e2f8*	*e2f*	E2F Transcription Factor 8	−1.32298	−2.06559	5.406993	−2.01843
gene27650:106566562	ENSGMOG00000000127	*snai1b*	*zf-c2h2*	snail family zinc finger 1	0.220371	−2.85429	5.304473	−2.67055
gene14139:106607389	ENSGMOG00000000115	*si:dkey-208k4.2*	*zf-c2h2*		0.193994	−1.89031	5.266383	−3.57007
gene29784:106568797	ENSGMOG00000002773	*mkxa*	*homeobox*	Homeodomain protein	0.302587	−1.29649	5.174461	−4.18055
gene36444:106575359	ENSGMOG00000020149	*si:dkeyp-113d7.1*	*zf-c2h2*		−1.49236	−1.6386	5.019571	−1.8886
gene32174:106571028	ENSGMOG00000011046	*fos*	*tf_bzip*	Fos Proto-Oncogene, AP-1 Transcription Factor Subunit	0.419622	−1.94431	4.658686	−3.134
gene11227:106604581	ENSGMOG00000016612	*lbx1a*	*homeobox*	ladybird homeobox 1a	−0.64393	−1.29966	4.654205	−2.71062
gene20791:106613765	ENSGMOG00000011735	*creb3l3a*	*tf_bzip*	cAMP responsive element binding protein 3-like 3a	−4.39031	−1.12504	−1.92737	7.442716
gene48589:106587347	ENSGMOG00000002967		*rxr-like*	The retinoid X receptor	−3.43146	−2.16225	−1.56866	7.162369
gene22207:106561422	ENSGMOG00000003773	*nr1h4*	*thr-like*	Nuclear Receptor Subfamily 1 Group H Member 4	−3.21863	−2.26679	−1.51679	7.002213
gene6072:106599177	ENSGMOG00000002344	*si:ch1073-291l11.1*	*zbtb*		−2.5624	−2.08568	−2.3124	6.960481
gene22802:106561958	ENSGMOG00000007809	*znf408*	*zf-c2h2*	Zinc Finger Protein 408	−2.94014	−2.30503	−1.49948	6.744649
gene31376:106570251	ENSGMOG00000000984	*nr0b2a*		Nuclear receptor subfamily 0, group B, member 2a	−2.77805	−2.31892	−1.47895	6.57592
gene48566:106587325	ENSGMOG00000002967		*rxr-like*	The retinoid X receptor	−3.19692	−0.82396	−2.44692	6.467793
gene52087:106590549	ENSGMOG00000001972	*hnf4g*	*rxr-like*	Hepatocyte Nuclear Factor 4 Gamma	−2.72588	−1.74915	−1.82963	6.304663
gene33755:106572609	ENSGMOG00000011421	*hnf4a*	*rxr-like*	Hepatocyte Nuclear Factor 4 Alpha	−2.33726	−2.04478	−1.83726	6.219307
gene27953:106566843	ENSGMOG00000015966	*nr1h5*	*thr-like*	Nuclear receptor subfamily 1, group H, member 5	−4.12551	−0.64098	−1.3563	6.12279

*The red-blue spectrum represents the scaled mean expression values*.

Ovary-specific genes were enriched for two GO-CC terms *nucleolus* and *integral component of membrane* (Table S5) and one GO-MF term *isomerase activity* (Table 1). The drivers of enrichment included hormone receptors such as *follicle stimulating hormone receptor, growth hormone receptor, trans-membrane proteins* members of solute carrier family and *3-oxo-5-alpha-steroid 4-dehydrogenase*. The list of highly expressed genes in the ovary is shown in Figure 4 and Table S2. The list of highly expressed TFs identified in the ovary (Table 2) included *e2f8, fos, kdm5ba, arid3b*, two Homeodomain proteins (*lbx1a* and *mkxa*) and two zinc finger proteins (*snai1b* and *si:dkey-208k4.2*).

The final tissue investigated was the liver, which had enriched GO terms in all three categories that included *lipid transport, lipoprotein metabolic process, extracellular region, extracellular space*, and *lipid binding* (Table 1; Table S6). The *lipid transport* term was the most highly over-represented (51-fold) based on genes coding for lipid transport-proteins (apolipoproteins). Other notable genes expressed primarily in the liver included coagulation factor, insulin-like growth factor-binding protein, peptide hormones, and genes with immune-related functions. The list of highly expressed genes in the liver (Figure 4; Table S2) included *serum albumins* (gene ID = gene1886:100136575, gene38403:100136922), *eggshell protein* (gene ID = gene22108:100136930), and *saxitoxin and tetrodotoxin-binding protein 1-like* (gene ID = gene18707:106611802). The list of highly expressed TFs identified in the liver (Table 2) included *creb3l3a*, nuclear receptors (*nr1h4, nr0b2a, nr1h5*), and hepatocyte nuclear factors (*hnf4g, hnf4a*).

## Discussion

Identifying the genes expressed in cells of a given tissue represents important baseline information essential for understanding tissue function and physiology more broadly. This study cataloged the collection of expressed genes in four salmon tissues, and documented significant changes that characterized tissues from each other. We identified the most abundant transcripts and the collection of genes and transcription factors (TFs) exhibiting tissue specific expression, and our findings were in broad agreement with expectation about the specific role of each organ. It is worthwhile noting that tissue specificity was considered only within the context of the tissues tested, and that examination of a more diverse collection of tissues may alter these findings. Nonetheless, the profile of each transcriptome captured GO enrichment terms consistent with expectation, and provided confidence that the dataset is of both high quality and useful for subsequent analysis. For example the enrichment of the GO terms *regulation of immunoglobulin secretion* and *extracellular region* derived from the presence of genes coding for pituitary hormones. Similarly in the ovary the enrichment of the GO term *integral component of membrane* derived from the presence of many hormone receptors such as follicle stimulating hormone receptor and growth hormone receptor which aligns with functions of the ovary. The identified signals with high expression reflect the physiological function of each tissue. For example, the high expression of the pituitary hormone *somatolactin beta* and *pro-opiomelanocortin* (*pomc*) was observed in the pituitary gland. Similarly the *pomc* gene is expressed in both the anterior and intermediate lobes of the pituitary gland. The same is true for other tissues as expression of genes encoding neuropeptides in the brain and genes encoding liver proteins such as albumin and eggshell proteins in the liver.

The dataset adds to the increasing volume of RNA-Seq information being made available by the research community. The majority of existing Atlantic salmon RNA-Seq datasets have been generated to address specific biological questions, including host response to disease challenge (Dettleff et al., 2017; Valenzuela-Muñoz et al., 2017; Rozas-Serri et al., 2018), developmental progression (Wang et al., 2014) or nutritional requirements (Jin et al., 2018). The consequence is a paucity of transcriptomic profiling of animals not subject to an experimental regime designed to elicit specific responses or that characterize a biological extreme. The survey sequencing performed here can therefore be considered baseline data, making it applicable to general studies seeking to understand normal state expression levels across tissues. Importantly, when co-analyzed with the other datatypes identifying gene regulatory elements inside FAASG, we anticipate utility for at least three experimental applications. Firstly, for interpretation of GWAS to prioritize candidate genes on the basis of their tissue specific expression in relation to the biology of the trait under investigation. Secondly, it will assist assigning putative function to salmonid specific genes without annotation using the “guilt by association” principal. Specifically, the function of uncharacterized genes can be deduced from their tissue specific co-expression with genes of known function. Finally, we anticipate the data to contribute to characterization of transcriptomic complexity that includes expression of transcript isoforms and temporal variation in expression that can only be addressed by extensive tissue sampling across multiple life history time points.

## Author contributions

AM, HK, JK, and BE conceived the study and contributed to the experimental design. HK and BE performed animal management and AM and JK contributed to sample acquisition. AM generated and analyzed the RNA-Seq data. TR contributed to the analysis. AM and JK drafted the manuscript which all authors contributed to.

### Conflict of interest statement

BE was employed by company Tassal. The remaining authors declare that the research was conducted in the absence of any commercial or financial relationships that could be construed as a potential conflict of interest.
