# Supplementary material for: Multi-Tissue Transcriptome Profiling of North American Derived Atlantic Salmon
Source: Front Genet. 2018 Sep 13;9:369. doi: 10.3389/fgene.2018.00369 (PMC6146974; doi:10.3389/fgene.2018.00369)
Supplement: Supplementary file 1 [file Data_Sheet_1.doc]

**Supporting Information**

**Multi-tissue transcriptome profiling of North American derived Atlantic salmon**

Amin Mohamed^1,4^, Harry King^2^, Brad Evans^3^, Antonio Reverter^1^ and James Kijas^1*^

Affiliations:

^1^CSIRO Agriculture and Food, Queensland Bioscience Precinct, St Lucia, QLD 4067, Australia

^2^CSIRO Agriculture, CSIRO, Hobart, Tasmania, Australia

^3^Tassal Ltd., Hobart, Tasmania, Australia

^4^Zoology Department, Faculty of Science, Benha University, Benha 13518, Egypt

Correspondence:

Dr James Kijas: [james.kijas@csiro.au](mailto:james.kijas@csiro.au)

Figure S1 A multidimensional scaling (MDS) plot produced by the plotMDS function in edgeR showing relationship among all replicates of pituitary gland (P_1:4), brain (B_1:4), ovary (O_1:4) and liver (L_1:4) samples. The distances shown are the biological coefficient of variation (BCV) between samples for the 500 genes that best distinguish the samples.

Figure S2 Level of agreement among the biological replicates of the four salmon tissues. The heat map shows the hierarchically clustered Spearman correlations resulting from comparing normalized expression for all samples against one another. Sample clustering indicates the consistency between the biological replicates of each of the four tissues.

Table S1. Illumina sequencing statistics. Data yield, number of obtained raw paired-end reads, number of mapped fragments for each RNA-Seq library.

| **Tissue** | **RNA-Seq library** | **Description** | **Data Yield (Gb)** | **Paired-end reads (M)** | **Mapped Reads (M)** |
| --- | --- | --- | --- | --- | --- |
| **Ovary** | Ovary_1 | Ovary Library biol. Rep. 1 | 12.53 | 41 | 31 |
|  | Ovary_2 | Ovary Library biol. Rep. 2 | 11.99 | 39 | 30 |
|  | Ovary_3 | Ovary Library biol. Rep. 3 | 10.8 | 35 | 25 |
|  | Ovary_4 | Ovary Library biol. Rep. 4 | 11.68 | 38 | 28 |
| **Pituitary** | Pituitary_1 | Pituitary Library biol. Rep. 1 | 11.77 | 38 | 30 |
|  | Pituitary_2 | Pituitary Library biol. Rep. 2 | 10.64 | 35 | 25 |
|  | Pituitary_3 | Pituitary Library biol. Rep. 3 | 11.79 | 39 | 28 |
|  | Pituitary_4 | Pituitary Library biol. Rep. 4 | 11.18 | 37 | 26 |
| **Brain** | Brain_1 | Brain Library biol. Rep. 1 | 10.17 | 33 | 23 |
|  | Brain_2 | Brain Library biol. Rep. 2 | 23.96 | 79 | 50 |
|  | Brain_3 | Brain Library biol. Rep. 3 | 11.6 | 38 | 26 |
|  | Brain_4 | Brain Library biol. Rep. 4 | 10.96 | 36 | 25 |
| **Liver** | Liver_1 | Liver Library biol. Rep. 1 | 12.05 | 39 | 21 |
|  | Liver_2 | Liver Library biol. Rep. 2 | 11.2 | 37 | 25 |
|  | Liver_3 | Liver Library biol. Rep. 3 | 10.51 | 34 | 24 |
|  | Liver_4 | Liver Library biol. Rep. 4 | 10.3 | 34 | 24 |
| **Mean** |  |  | 12.1 | 40 | 28 |

Table S2. Top 10 highly expressed gene lists and their putative functions in each tissue

| **Gene ID** | **Annotation** | **Function** |
| --- | --- | --- |
| **Brain cluster** | | |
| **gene43872:106582708** | *Synaptotagmin 2-like* | Synaptic vesicle-associated membrane protein |
| **gene48716:106587477** | *Vesicular glutamate transporter 2.1* | Transmission of Gonadotophin-Releasing Hormone |
| **gene2585:106566461** | *Nuerofilament light polypeptide-like* | Maintenance of neuronal caliber |
| **gene945:106603349** | *Visinin-like protein 1* | Neuronal calcium sensor protein |
| **gene29879:106568768** | *Ras-related protein Rab-6 B-like* | Retrograde transport in neuronal cells |
| **gene24010:100306872** | *Neurofilament medium polypeptide* | Maintenance of neuronal caliber |
| **gene24009:106563330** | *Neurofilament light polypeptide-like* | Maintenance of neuronal caliber |
| **gene45936:106584654** | *Creattine kinase S-type, mitochondrial* | Maintenance of energy homeostasis |
| **gene39762:106578701** | *Glucagon family neuropeptides-like* | Neuropeptide in the brain |
| **gene18285:100196798** | *Visinin-like protein 1* | Neuronal calcium sensor protein |
| ***Pituitary cluster*** | | |
| **gene24503:106563665** | *Ependymin-like* | Calcium ion binding |
| **gene14850:100169856** | *Pro-opiomelanocortin B* | Hormone activity |
| **gene10336:100196589** | *Somatolactin beta* | Hormone activity |
| **gene42357:100136491** | *Somatolactin beta* | Hormone activity |
| **gene43413:106582253** | *Neuromedin U-like* | Signal peptide |
| **gene18355:100510783** | *Pro-opiomelanocortin A1* | Hormone activity |
| **gene139:100510784** | *Pro-opiomelanocortin A1* | Hormone activity |
| **gene29566:106568494** | *Nose resistant to fluoxetine protein 6-like* | Transport |
| **gene6704:106600448**  **gene2482:100192333** | *Somatostatin receptor type 3-like*  *LIM homeobox 3 (lhx3)* | G-protein coupled receptor activity  Sequence-specific DNA binding |
| ***Liver cluster*** | | |
|  |  |  |
| **gene11192:106604524** | *Tryptophan 2,3-dioxygenase* | Catabolism of tryptophan |
| **gene31979:106570873** | *Sonic hedgehog protein-like* | Development |
| **gene21398:106560589** | *Histidine-rich glycoprotein-like* | Liver-derived plasma glycoprotein |
| **gene52069:106590538** | *Lipocalin-like* | Transporter activity |
| **gene1886:100136575** | *Serum albumin 1* | Plasma protein |
| **gene29410:106568325** | *Trypsin inhibitor CITI-1-like* | Enzyme inhibtion |
| **gene14548:106607754** | *Protein LEG1 homolog* | Liver development |
| **gene18707:106611802** | *Saxitoxin and tetradotoxin-binding protein 1-like* | Toxin accumulation and/or excretion |
| **gene22108:100136930** | *Eggshell protein* | Vitellogenesis |
| **gene38403:100136922** | *Serum albumin 2* | Plasma protein |
| ***Ovary cluster*** | | |
| **gene29458:106568419** | *Cryptic protein-like* | Signal peptide |
| **gene29309:106568163** | *Protein phosphatase 1 regulatory subunit 3B* | Glycogen metabolism |
| **gene16056:106609093** | *Protein DENND6B-like* | Rab-mediated trafficking pathways |
| **gene22941:106562137** | *Cyclin-B1-like* | Cell division |
| **gene36634:106575547** | *Sodium/myo-inositol cotransporter-like* | Transporter activity |
| **gene24560:106563668** | *Putative bifunctional UDP-N-acetylglucosamine transferase and deubiquitinase ALG13* | Transferase activity |
| **gene24707:106563854** | *Cyclin A1* | Control of meiosis |
| **gene2120:106561951** | *Cathepsin L1-like* | Cysteine proteinase activity |
| **gene49943:106588276** | *alpha-2,8-sialytransferase 8F-like* | Glycodyltransferase activity |
| **gene51482:106589930** | *leucine-rich repeat, immunoglobulin-like domain and transmembrane domain-containing protein 2* | Transmembrane protein |

Table S3. Annotations of the genes of the GO-BP *regulation of immunoglobulin secretion* and GO-CC extracellular region enriched among pituitary gland-specific genes

| ID | ***GO-BP regulation of immunoglobulin secretion*** ***genes*** |
| --- | --- |
| 100136588 | *growth hormone pre-peptide* |
| 100136580 | *prolactin* |
| 106607462 | *somatotropin-2* |
| *ID* | **GO-CC *extracellular region*** **genes** |
| 100286517 | *glycoprotein hormones alpha chain 1(glha1)* |
| 100380386 | *gonadotropin subunit beta-2(gthb2)* |
| 100380514 | *corticotropin releasing hormone binding protein (crhbp)* |
| 100136440 | *pituitary alpha-2 glycoprotein hormone subunit precursor (glha2)* |
| 100510783 | *pro-opiomelanocortin A1(LOC100510783)* |
| 100510784 | *pro-opiomelanocortin A2(LOC100510784)* |
| 100136580 | *Prolactin (LOC100136580)* |
| 100169856 | *proopiomelanocortin B (LOC100169856)* |
| 100196589 | *somatolactin beta (LOC100196589)* |
| 100136491 | *somatolactin (LOC100136491)* |
| 100136355 | *thyroid stimulating hormone, beta subunit (tshb)* |

Table S4. Annotations of the genes of GO-CC *extracellular region* and GO-MF *ion binding* enriched among brain-specific genes

| IDs | ***GO-CC extracellular region genes*** |
| --- | --- |
| 100196096 | *C-C motif chemokine 8 (ccl8)* |
| 100196313 | *C-type natriuretic peptide 1 (anfc1)* |
| 100306726 | *C-type natriuretic peptide 3 (anfc3)* |
| 100194948 | *CEF-10 (cef10)* |
| 100194898 | *glucagon family neuropeptides (paca)* |
| 100196250 | *metalloproteinase inhibitor 2 (timp2)* |
| 100196110 | *progonadoliberin-2 (gon2)* |
| 100195758 | *stromal cell-derived factor 1 (sdf1)* |
| 106587496 | *calcitonin-1-like (LOC106587496)* |
| 100286641 | *calcitonin/calcitonin-related polypeptide, alpha (calca)* |
| 100194429 | *cholecystokinin-L (cck-l)* |
| 100194430 | *cholecystokinin-N (cck-n)* |
| 106605490 | *cholecystokinin-like (LOC106605490)* |
| 106579547 | *corticoliberin-1-like (LOC106579547)* |
| 100195676 | *fibroblast growth factor 3 (fgf3)* |
| 100136397 | *myostatin 1a (gdf-8)* |
| 100136531 | *myostatin 1b (gdf-8)* |
| 100286784 | *neuropeptide Y (npy)* |
| 100194431 | *peptide YY (pyy)* |
| 100196814 | *proenkephalin (penk)* |
| 100196143 | *thyrotropin-releasing hormone (trh)* |
| IDs | *GO-MF ion binding genes* |
| 100194596 | *aspartyl beta-hydroxylase-like (LOC100194596)* |
| 100286515 | *EF-hand domain family, member D1 (efhd1)* |
| 100196805 | *myosin regulatory light chain 2b, cardiac muscle isoform (mlrb)* |
| 100195117 | *nel protein (nel)* |
| 100380479 | *parvalbumin alpha (prva)* |
| 100196669 | *s100 calcium binding protein v2-like (loc100196669)* |
| 100286437 | *s100 calcium binding protein, beta (neural)(s100b)* |
| 100196798 | *visinin-like protein 1 (visl1)* |
| 106611171 | *calmodulin 1 (calm1)* |
| 106603160 | *calmodulin (loc106603160)* |
| 106612378 | *calmodulin (loc106612378)* |
| 106560817 | *calretinin-like (loc106560817)* |
| 106573754 | *calretinin-like (loc106573754)* |
| 106574292 | *protein s100-b-like (loc106574292)* |
| 100380447 | *sarcoglycan, epsilon (sgce)* |
| 106571909 | *troponin c, slow skeletal and cardiac muscles (loc106571909)* |
| 106603349 | *visinin-like protein 1 (loc106603349)* |

Table S5. Annotations of the genes of the GO-CC *integral* *component of membrane* enriched among ovary-specific genes

| ID | Gene Name |
| --- | --- |
| 100196185 | *3-oxo-5-alpha-steroid 4-dehydrogenase 2(s5a2)* |
| 100194793 | *anti-apoptotic protein nr13(nr13)* |
| 100196057 | *apoptosis regulator bcl-x(bclx)* |
| 100286692 | *CD032 protein(cd032)* |
| 106586182 | *CD302 antigen-like(LOC106586182)* |
| 100196519 | *CF105 protein(cf105)* |
| 100195338 | *choline transporter-like protein 2(ctl2)* |
| 100306741 | *claudin-like protein zf4a22(cldx)* |
| 100194791 | *DnaJ homolog subfamily C member 1(dnjc1)* |
| 100195085 | *DnaJ homolog subfamily C member 11(dcj11)* |
| 100196876 | *E3 ubiquitin-protein ligase MARCH2(marh2)* |
| 100195143 | *E3 ubiquitin-protein ligase RNF128(rn128)* |
| 100286454 | *elongation of very long chain fatty acids protein 1(elov1)* |
| 100196617 | *estradiol 17-beta-dehydrogenase 12-b(dh12b)* |
| 100141317 | *fxyd domain containing ion transport regulator 5a(fxyd5a)* |
| 100196506 | *gli pathogenesis related 1(glipr1)* |
| 100380444 | *heparan sulfate 2-o-sulfotransferase 1(hs2st)* |
| 100195516 | *integral membrane protein gpr137(g137a)* |
| 100195064 | *interleukin-4 receptor alpha chain(il4ra)* |
| 100380426 | *interleukin-6 receptor subunit alpha(il6ra)* |
| 100286474 | *lysozyme g(lyg)* |
| 100286625 | *marvel domain-containing protein 3(mald3)* |
| 100380416 | *matrix-remodeling-associated protein 8(mxra8)* |
| 100195297 | *monocyte to macrophage differentiation protein(paqrb)* |
| 106612446 | *nadph--cytochrome p450 reductase(loc106612446)* |
| 106572490 | *orm1-like protein 2(loc106572490)* |
| 100195699 | *p2y purinoceptor 8(p2ry8)* |
| 100196500 | *phs1 protein(phs1)* |
| 100194818 | *phosphatidic acid phosphatase type 2 domain-containing protein 1b(ppc1b)* |
| 100196322 | *programmed cell death 1 ligand 1(pdl1)* |
| 100195488 | *proteinase-activated receptor 2(par2)* |
| 106571628 | *saysvfn domain-containing protein 1-like(loc106571628)* |
| 100136398 | *st2l protein-like(st2)* |
| 100196049 | *signal peptidase complex catalytic subunit sec11a(sc11a)* |
| 100380572 | *sodium-dependent proline transporter(sc6a7)* |
| 100380846 | *solute carrier family 22 member 18(s22ai)* |
| 100194825 | *solute carrier family 25 member 40(s2540)* |
| 100380302 | *solute carrier family 41 member 2(s41a2)* |
| 100195408 | *sphingomyelin phosphodiesterase 4(nsma3)* |
| 100380840 | *star-related lipid transfer protein 3(star3)* |
| 106582802 | *t-cell leukemia translocation-altered gene protein homolog(loc106582802)* |
| 106575093 | *t-cell-specific surface glycoprotein cd28-like(loc106575093)* |
| 100194983 | *tetraspanin-14(tsn14)* |
| 100196850 | *translocon-associated protein subunit delta(ssrd)* |
| 100195591 | *transmembrane 6 superfamily member 1(tm6s1)* |
| 100286528 | *transmembrane protein 179b(t179b)* |
| 100380433 | *transmembrane protein 184a(t184a)* |
| 100196417 | *transmembrane protein 188(tm188)* |
| 100195683 | *transmembrane protein 70(tmm70)* |
| 100196431 | *tumor-associated calcium signal transducer 2(tacd2)* |
| 106613831 | *udp-n-acetylglucosamine transporter-like(loc106613831)* |
| 100380310 | *unc93-like protein mfsd11(mfs11)* |
| 100196791 | *vacuolar proton translocating atpase 116 kda subunit a(vpp3)* |
| 100194742 | *zinc transporter slc39a7(ke4)* |
| 100195485 | *abhydrolase domain containing 12(abhd12)* |
| 100195303 | *ancient ubiquitous protein 1(aup1)* |
| 100380342 | *angiotensin ii receptor type 2(agtr2)* |
| 100195122 | *brain protein i3(bri3)* |
| 106607447 | *cathepsin d-like(loc106607447)* |
| 106572113 | *cytochrome c oxidase subunit 4 isoform 2, mitochondrial-like(loc106572113)* |
| 106605225 | *cytochrome c oxidase subunit 7a-related protein, mitochondrial(loc106605225)* |
| 106600687 | *dehydrogenase/reductase 7b(dhrs7b)* |
| 100380525 | *ferric-chelate reductase 1(frrs1)* |
| 100135779 | *follicle stimulating hormone receptor(fsh-r)* |
| 106610304 | *group xiia secretory phospholipase a2-like(loc106610304)* |
| 100136463 | *growth hormone receptor isoform 2 precursor(loc100136463)* |
| 100136401 | *interleukin 1 receptor accessory protein(il-1racp)* |
| 106581741 | *interleukin-10 receptor subunit beta-like(loc106581741)* |
| 100196773 | *lipolysis stimulated lipoprotein receptor(lsr)* |
| 106607342 | *lipopolysaccharide-induced tumor necrosis factor-alpha factor homolog(loc106607342)* |
| 100196786 | *major facilitator superfamily domain containing 1(mfsd1)* |
| 100196291 | *mal, t-cell differentiation protein(mal)* |
| 100195434 | *mannosyl (alpha-1,6-)-glycoprotein beta-1,2-n-acetylglucosaminyltransferase(mgat2)* |
| 101448023 | *mitochondrial ubiquitin ligase activator of nf-kb(mulan)* |
| 106587574 | *nuclear envelope phosphatase-regulatory subunit 1(loc106587574)* |
| 106577458 | *oligosaccharyltransferase complex subunit ostc-like(loc106577458)* |
| 100846964 | *osteopetrosis associated transmembrane protein 1(ostm1)* |
| 106565535 | *peptidyl-prolyl cis-trans isomerase fkbp11-like(loc106565535)* |
| 106589625 | *peripheral myelin protein 22(pmp22)* |
| 100380567 | *peroxisome biogenesis factor 13(pex13)* |
| 100196273 | *phosphatidylethanolamine n-methyltransferase(pemt)* |
| 106584151 | *prolactin regulatory element-binding protein-like(loc106584151)* |
| 106606980 | *protein fam195b-like(loc106606980)* |
| 106561292 | *protein fam3c-like(loc106561292)* |
| 106585762 | *protein kish-a-like(loc106585762)* |
| 106574520 | *protein transport protein sec61 subunit beta-like(loc106574520)* |
| 100306734 | *protein-o-mannose kinase(pomk)* |
| 100195280 | *rhomboid domain containing 1(rhbdd1)* |
| 100136503 | *scavenger receptor class b type i(sr-bi)* |
| 100195574 | *sideroflexin 2(sfxn2)* |
| 106562465 | *signal peptidase complex catalytic subunit sec11a(loc106562465)* |
| 100195653 | *signal peptidase complex subunit 3 homolog (s. cerevisiae)(spcs3)* |
| 106602378 | *signal peptidase complex subunit 3(loc106602378)* |
| 106565345 | *sodium- and chloride-dependent gaba transporter 2-like(loc106565345)* |
| 100195099 | *solute carrier family 35 member a4(slc35a4)* |
| 106580656 | *stress associated endoplasmic reticulum protein 1(serp1)* |
| 106568929 | *stress-associated endoplasmic reticulum protein 1-like(loc106568929)* |
| 106567022 | *surfeit locus protein 4-like(loc106567022)* |
| 106566367 | *synaptophysin-like protein 1(loc106566367)* |
| 106605215 | *tetraspanin-13-like(loc106605215)* |
| 106602560 | *toll like receptor 3(tlr3)* |
| 100306868 | *translocation-associated membrane protein 1-like 1(tr1l1)* |
| 106570196 | *translocon-associated protein subunit beta-like(loc106570196)* |
| 100195239 | *transmembrane emp24 protein transport domain containing 9(tmed9)* |
| 106601270 | *transmembrane protein 106b-like(loc106601270)* |
| 100196498 | *transmembrane protein 126a(tmem126a)* |
| 100380489 | *transmembrane protein 129(tmem129)* |
| 106569654 | *transmembrane protein 14c-like(loc106569654)* |
| 100380459 | *transmembrane protein 43(tmem43)* |
| 100195134 | *transmembrane protein adipocyte associated 1(tpra1)* |
| 100195187 | *transport protein sec61 subunit alpha(s61a1)* |
| 100196587 | *transport protein sec61 subunit beta(sc61b)* |
| 100195609 | *zinc finger dhhc-type containing 4(zdhhc4)* |
| 106613589 | *zinc transporter 7-like(loc106613589)* |
| 106561904 | *zinc transporter zip1-like(loc106561904)* |

Table S6. Annotations of the genes of the GO-BP *lipid transport,* GO-CC *extracellular region* and GO-MF *lipid binding* enriched among liver-specific genes

| ID | ***GO-BP lipid transport genes*** |
| --- | --- |
| 100136573 | *apolipoprotein A-I(LOC100136573)* |
| 100196111 | *apolipoprotein A-I(apoa1)* |
| 106605692 | *apolipoprotein A-IV-like(LOC106605692)* |
| 106605287 | *type-4 ice-structuring protein LS-12-like(LOC106605287)* |
| ID | **GO-CC *extracellular region* genes** |
| 100380442 | *coagulation factor IX(fa9)* |
| 100136551 | *IGF binding protein 3(igfbp-2b1)* |
| 100196111 | *apolipoprotein A-I(apoa1)* |
| 106605692 | *apolipoprotein A-IV-like(LOC106605692)* |
| 100195639 | *c-reactive protein, pentraxin-related(crp)* |
| 106584587 | *hepcidin-1(LOC106584587)* |
| 106590538 | *lipocalin-like(LOC106590538)* |
| 100136583 | *pentraxin(LOC100136583)* |
| 106561522 | *protein AMBP-like(LOC106561522)* |
| 100195744 | *retinol binding protein 4(rbp4)* |
| 100136400 | *secreted phosphoprotein 2(spp2)* |
| 106605287 | *type-4 ice-structuring protein LS-12-like(LOC106605287)* |
| ID | **GO-MF *lipid binding* genes** |
| 100136573 | *apolipoprotein A-I(LOC100136573)* |
| 100196111 | *apolipoprotein A-I(apoa1)* |
| 106605692 | *apolipoprotein A-IV-like(LOC106605692)* |
| 100136575 | *serum albumin 1(alb1)* |
| 100136922 | *serum albumin 2(LOC100136922)* |
| 106605287 | *type-4 ice-structuring protein LS-12-like(LOC106605287)* |
